# Identification of *Pseudomonas protegens* and *Bacillus subtilis* Antimicrobials for Mitigation of Fuel Biocontamination

**DOI:** 10.3390/biom15020227

**Published:** 2025-02-04

**Authors:** Amanda L. Barry Schroeder, Adam M. Reed, Osman Radwan, Loryn L. Bowen, Oscar N. Ruiz, Thusitha S. Gunasekera, Andrea Hoffmann

**Affiliations:** 1 Environmental Microbiology, Fuels & Combustion Division, University of Dayton Research Institute, Dayton, OH 45469, USA; adam.reed@udri.udayton.edu (A.M.R.); loryn.bowen@udri.udayton.edu (L.L.B.); 2 Power & Energy Division, University of Dayton Research Institute, Dayton, OH 45469, USA; osman.radwan@udri.udayton.edu; 3 Biomaterials Branch, Photonic, Electronic & Soft Materials Division, Materials and Manufacturing Directorate, Air Force Research Laboratory, Wright-Patterson Air Force Base, OH 45433, USA; oscar.ruiz@us.af.mil; 4 Fuels & Energy Branch, Aerospace Systems Directorate, Air Force Research Laboratory, Wright-Patterson Air Force Base, OH 45433, USA; thusitha.gunasekera.1@us.af.mil

**Keywords:** biocontamination of hydrocarbon fuels, fuel-derived microbial isolate repository, antimicrobial screening, agar plug screening, biocontrol culture filtrate testing, Jet A fuel, pyochelin, *Gordonia* sp., aviation fuel sustainment

## Abstract

Hydrocarbon fuel biofouling and biocorrosion require expensive cleanup of aviation infrastructures unless appropriate sustainment measures are applied. The identification of novel biological control agents offers promising alternatives to the current chemical biocides used in fuel sustainment. In this study, 496 microbial fuel isolates from our in-house repository were screened to identify new endogenously produced antimicrobial compounds. Using agar plug screening, liquid culture growth testing, and Jet A fuel culture assays, the two fuel-isolate strains *Pseudomonas protegens* #133, and *Bacillus subtilis* #232 demonstrated promising biocontrol activity against bacteria, yeast, and filamentous fungi. Liquid chromatography-quadrupole time of flight tandem mass spectrometry (LC-QTOF-MS/MS) of #232 culture filtrate identified several common lipopeptide antimicrobials including gageostatin C, gageopeptin B, and miscellaneous macrolactins. In contrast, LC-QTOF-MS/MS identified the siderophore pyochelin as one of the predominant compounds in #133 culture filtrate with previously demonstrated antimicrobial effect. Jet fuel microbial consortium culture testing of #133 culture filtrate including flow-cytometry live/dead cell mechanism determination demonstrated antimicrobial action against Gram-positive bacteria. The study concludes that antimicrobial compounds secreted by #133 have bactericidal effects against *Gordonia* sp. and cause cell death through bacterial lysis and membrane damage with potential applications in the biocidal treatment of hydrocarbon-based aviation fuels.

## 1. Introduction

Present fuel system infrastructure sustainment efforts require frequent fuel quality and contaminant testing to prevent clogging of fuel lines and the replacement of fuel engineering components that are affected by biofouling and bio-based corrosion [1,2,3]. The identification of specialized microbes that can use jet fuel hydrocarbons as a substrate and consequential studies that focus on microbial metabolism and biofilm formation give insight into potential antimicrobial treatment options [3,4,5,6]. For instance, microbes can achieve antimicrobial resistance via multiple biochemical pathways, including the metabolic adjustment of solvent tolerance via efflux pump activation and the initiation of biofilm formation [5,7,8,9,10]. In previous efforts, our group tested the use of small-molecule efflux pump blockers in the treatment of fuel microbial contamination. This research effort tries to identify natural biocontrol compounds that are intrinsically produced by microbes and can be applied along with efflux pump inhibitors as a combinatorial strategy to overcome microbial resistance in fuel tanks.

Laboratory screening for novel antimicrobials has utilized the competitive nature of environmental consortium microbes where nutrient-competing strains produce inhibitory compounds to block other competitors [11,12,13,14]. One class of bacterial and fungal biocides with relatively higher stability includes antimicrobial peptides (AMPs) [13,15]. The mechanism of action of most AMPs is either through inhibition of cell division by causing intracellular toxicity or through exterior membrane permeabilization [13]. Recently, our group demonstrated the application of sheep myeloid antimicrobial peptide (SMAP-18) and synthetic pyochelin in the treatment of fuel biocontamination [16].

In this study, 496 microbial hydrocarbon fuel isolate strains from our in-house repository were tested by agar plug screening to identify potential naturally produced biocontrol compounds that can be utilized as biocides against other fuel systems’ contaminating microbes. Agar plug diffusion screening represents a common and well-established method to screen for antimicrobial activity throughout the microbiology community [17,18]. To distinguish between potential cell-to-cell interactions and antimicrobial compound-dependent growth inhibition, we established a liquid culture filtrate screening assay that ensured the activity measurement of secreted soluble biocontrol compounds. Using these methods, two fuel-isolate strains, *Pseudomonas protegens* #133 and *Bacillus subtilis* #232, demonstrated promising biocontrol activity against Gram-positive and Gram-negative bacteria, yeast, and filamentous fungi. According to previous studies, *B. subtilis* is known to produce over 200 antimicrobial compounds, including bacillaene, bacilysin, and surfactin [19]; lipopeptides, such as gageostatins and gageopeptins; [20,21,22] iturins and fengycins [19,23]; polyketide macrolactins [20,24,25]; and volatile organic compounds (VOCs) with antifungal properties [23,26,27]. In contrast, most of the antimicrobial compounds produced by *P. protegens* include small molecules and lipid compounds, such as rhamnolipids, and quorum-sensing molecules, including pyoverdine, pyocyanin, and pyochelin [28]. By using LC-QTOF-MS/MS, this study gives a detailed insight into the compounds specifically contained in culture filtrates of isolates #133 and #232. To further test the efficiency of #133 culture filtrate in hydrocarbon fuels, this study utilized Jet A fuel microbial consortium culture, a common method used by the fuel testing community [6,16,29,30], as a tool to simulate microbial growth as it happens in a fuel tank environment. By using the Jet A fuel culture of *Gordonia* sp., the antimicrobial mode of action of #133 culture filtrate was determined by flow cytometry (FCM) live/dead SYTO9/Propidium iodide (PI) dye exclusion assay. This FCM-based method was chosen since it allows real-time assessment of cell viability and the antimicrobial agent-dependent appearance of cytotoxicity [31,32,33]. The subsequent findings show promising results for the use of #133 crude culture filtrate as a biocontrol agent for Gram-positive bacteria, with notable bactericidal efficacy against the fuel contaminant *Gordonia* sp., whereas *B. subtilis* #232, with few exceptions, was more efficient against fungal targets.

## 2. Material and Methods

### 2.1. Materials

Pyochelin I/II was obtained from Santa Cruz Biotechnologies (Cat# sc-506665; Santa Cruz Biotechnologies, Santa Cruz, CA, USA). The filamentous fungus *Hormoconis resinae* (ATCC 22711), the yeast *Yarrowia lipolytica* (ATCC 20496), and the Gram-positive bacterium *Gordonia* sp. (ATCC BAA-559) were obtained from the American Type Culture Collection (ATCC) (Manassas, VA, USA). The Gram-negative bacterium *Pseudomonas aeruginosa* PAO1 represents a laboratory strain (taxonomy ID 208964.1). The Gram-negative bacteria *Escherichia coli* Jm109 were obtained from Promega (Cat# L2005; Promega, Madison, WI, USA). The chosen fuel test strains included the Gram-negative bacteria *Pseudomonas putida, Acinetobacter venetianus, and Hydrocarboniphaga effusa*; the Gram-positive bacteria *Rhodococcus equi*, *Nocardioides albus*, and *Bacillus atrophaeus*; the yeasts *Meyerozyma guilliermondii* and *Candida ethanolica*; and the filamentous fungi *Aspergillus versicolor* and *Fusarium oxysporum* were obtained from the Air Force Research Laboratory (AFRL) microbial repository, isolated from bio-contaminated fuel samples.

### 2.2. Agar Plug Diffusion Testing

Donor and recipient test microbes were plated on agar culture plates with growth medium, e.g., tryptic soy agar (TSA, REF 236950, BD Difco, Rutherford, NJ, USA) or potato dextrose agar (PDA, REF 213400, BD Difco, Rutherford, NJ, USA), and incubated at 28 °C for 1–3 days. To generate plates, bacterial cells or fungal spores were directly suspended in the 50% agar medium at 1 × 10^6^ fungal spores or bacteria cells/mL as the final concentration to allow for the even distribution of microbes throughout the dried plate. Donor plates were punched with 5 mm sterile cork borers that were excised to produce agar plate plugs. The excised donor plugs were placed onto the recipient plate containing the test microbes. Following the addition of plugs, plates were incubated at 28 °C overnight. Control plates only received non-inoculated plugs. After incubation, the radius of inhibition zones from the control and treatment were measured in mm at 24, 48, 72, and 96 h after incubation.

Detailed agar plug screening process: (1) All 496 microbial fuel isolates were tested as agar donor plugs against *Pseudomonas putida* due to the high antimicrobial resistance of this strain. Out of 496 isolates, 89 demonstrated inhibition zones. (2) These 89 microbes were tested as donor plugs against one Gram-positive bacterium: *Gordonia* sp., one yeast: *Yarrowia lipolytica*, and one filamentous fungus: *Hormoconis resinae.* (3) The top 18 isolates with proven inhibition zones were tested as donor plugs against eight additional microbes, including the step (2) previously tested microbes, for a total of 12 microbes: *Acinetobacter venetianus*, *Hydrocarboniphaga effusa*, *Gordonia* sp., *Pseudomonas putida*, *Rhodococcus equi*, *Nocardioides albus*, *Yarrowia lipolytica*, *Meyerozyma guilliermondii*, *Candida ethanolica*, *Hormoconis resinae*, *Aspergillus versicolor*, and *Fusarium oxysporum.* (4) The top nine isolates were additionally tested as donor plugs against Gram-positive *Bacillus atrophaeus* and Gram-negative *Escherichia coli* Jm109 for a total of 14 microbes tested, including the step (3) previously tested microbes (Supplemental Data S0).

### 2.3. Purification of Culture Filtrates for Antimicrobial Testing

Isolates were pre-cultured from frozen stock and grown overnight at 28 °C on either tryptic soy agar (TSA) for bacterial or potato dextrose agar (PDA) for fungal cultures. The next day, one colony was transferred to a 20 mL liquid culture containing tryptic soy broth (TSB, REF 211825, BD Difco, Rutherford, NJ, USA) and incubated overnight under agitation (200 revolutions per minute, (RPM)) at 28 °C. For fungal cultures, the spores were scraped off at 72 h incubation and filtered with a 0.2 µm filter to only collect the spores, then steps were continued in a similar fashion. The next day, liquid cultures were washed three times with phosphate-buffered saline (PBS, BP2944-100, Fisher Scientific, Waltham, MA, USA) followed by serial dilution and incubation overnight at 28 °C for colony forming unit (CFU) determination. According to the CFU, 3 × 10^7^ cells/ mL of the isolate were added to 25 mL of M9 minimum medium (M9, M6030, Sigma Aldrich, St. Louis, MA, USA) supplemented with 0.5% glycerol (BP229-1, Fisher Scientific, Waltham, MA, USA). The samples were placed in a 28 °C shaking incubator at 200 RPM for 24–72 h. An amount of 1 mL of liquid culture was diluted and plated to obtain the CFU count of the isolate. To generate culture filtrates, cultures were spun down at 8500 RPM for 10 min and filtered with 0.2 µm of nylon filters (Corning Cat. # 431224). The absorbance of the culture filtrates was read at 205 and 254 nm in a Nanodrop (Nanodrop 2000c, Thermo Scientific, Waltham, MA, USA) instrument to obtain the protein, peptide, and lipid concentration. Prior to use in the experiments, an aliquot of culture filtrates was plated and assessed for growth to ensure the complete removal of microbial cells. For 96-well liquid culture testing, and fuel consortium culture testing, the #232 and #133 crude culture filtrates were dried down by nitrogen evaporation and re-diluted in the assay buffers with a stock concentration of 1 mg/mL. The reconstituted crude filtrate stock solutions were sterile filtered with a 0.2 µm filter and added to the experiments according to the given final concentrations at 25, 50, 100, or 500 µg/mL.

### 2.4. Antimicrobial Liquid Culture Screening Assay

Liquid media culture studies were performed in 96-well plates with 200 µL total volume, using either M9 minimum medium/0.5% glycerol (bacteria) or yeast nitrogen base without amino acids (YNB, REF 233520, BD Difco, Rutherford, NJ, USA)/0.5% glycerol (fungi), followed by inoculation with or without (medium control) the test microbial strains of Gram-negative bacteria, Gram-positive bacteria, yeast, and filamentous fungi at a final concentration of 1 × 10^4^ cells/mL, with at least three replicates. Cultures were supplemented with the corresponding test culture filtrate at 1:1 *v*/*v*, e.g., 100 µL containing a concentration of 1.38 mg/mL dry weight for isolate #133 or 1.96 mg/mL dry weight for isolate #232. The samples were incubated in the corresponding substrate for 14 days and samples were collected on day 0 and throughout the 14 days. To account for enhanced evaporation on the plate edge, samples were plated in the middle of the 96-well plate by leaving the edges unfilled prior to the addition of the clear sterile plate sealing membrane. Prior to and after sample collection, at the corresponding time points, plates were spun for 10 min at 3000 RPM to remove condensation. Serial dilutions were made prior to CFU culture plating, which were used to quantify the number of living cells in the presence of each concentration for each isolate.

### 2.5. Colony Forming Unit Determination

For CFU/mL determination, 200 µL of culture samples were collected, serially diluted, and plated onto agar media. Triplicates of the samples from each different time point were collected from each timepoint for CFU culture plating on TSA plates (bacteria) and PDA supplemented with ampicillin, kanamycin, and spectinomycin at 50 μg/mL (fungi) (Ampicillin, MT61238RM; Kanamycin, BP9065 BP29571; Spectinomycin; Fisher Scientific, Waltham, MA, USA). The plates were incubated at 28 °C overnight, followed by CFU counting. The replicates were either plated manually or by using an easySpiral Pro Plater (Interscience, Woburn, MA, USA), according to the manufacturer’s protocol. CFU/mL was either determined by counting colonies on manually spread plates or by using the automatic plate reader Scan 1200 Colony Counter (Interscience, Woburn, MA, USA), according to the manufacturer’s protocol.

### 2.6. Fuel Microbial Consortium Culture Assay

Assays for the fuel microbial consortium cultures were performed in 40 mL glass vials, using 20 mL Jet A fuel as the carbon source, underlain with 5 mL water bottom total containing minimal growth media in a 1:1 (*v*/*v*) mixture of M9 medium/YNB, or the #133 crude filtrate. Fuel cultures were inoculated with a microbial consortium of the Gram-negative bacteria *P. putida*, Gram-positive bacteria *Gordonia* sp., yeast *Y. lipolytica,* and filamentous fungi *H. resinae,* at a final concentration of 1 × 10^4^ cells/mL containing an equal amount of each microbe. The cultures were supplemented with the chosen antimicrobials, e.g., crude culture filtrate at doses of 0.0 µg/mL (microbial growth control) or administered at 100 µL (0.69 mg/ mL dry weight, dissolved as 0.276 mg/ mL); 500 µL (3.45 mg/ mL dry weight, dissolved as 1.38 mg/ mL); 1 mL (6.9 mg/ mL dry weight, dissolved as 2.76 mg/ mL); and 2.5 mL (17.25 mg/ mL dry weight, dissolved as 6.9 mg/ mL) of their total volume, followed by incubation at 28 °C without agitation. Samples of microbial consortia were taken over the course of 29 days. For each sample, 200 µL was collected across different time points and serial dilutions were made for CFU culture plating. Three technical samples were collected from each vial for CFU culture plating on agar plates, as described above (Section 2.5).

### 2.7. Culture Filtrate Compound Purification by Ethyl Acetate Lipid Extraction

For lipid extraction, microbes were grown for three days at a starting concentration of 3 × 10^7^ cells/mL in M9 minimum medium supplemented with 0.5% glycerol prior to sterile filtration with a 0.2 µm filter to obtain crude culture filtrates. For ethyl acetate lipid extraction, crude culture filtrates were acidified with 10 µL of 1N HCl, followed by an overnight incubation. In brief, ethyl acetate liquid–liquid extractions were performed in Corning Falcon (Cat. #1495949A, Fisher Scientific, Waltham, MA, USA) 50 mL vials using a 1:1 (*v*/*v*) ethyl acetate to culture filtrate ratio, followed by vortexing for 30 s and incubation for 5 min to accomplish phase separation. The upper organic ethyl acetate phase was carefully removed by not disturbing the interphase or the lower water phase using a serological pipet. Following the replenishment of the upper phase with fresh ethyl acetate, the extraction was repeated two more times. The resulting ethyl acetate extracts were combined and evaporated to dryness under nitrogen to obtain lipid extracts and re-diluted in the assay buffers with a stock concentration of 1 mg/mL. Experimental samples were supplemented with reconstituted crude filtrate stock solutions at final concentrations of 25, 50, 100, or 500 µg/mL. Lipid extracts were either analyzed directly by LC-QTOF-MS/MS or fractionated by semi-preparative high-performance liquid chromatography (semi-prep HPLC).

### 2.8. Culture Filtrate Compound Purification by Fractionation Using Semi-Prep High-Performance Liquid Chromatography (HPLC)

For semi-prep HPLC (UltiMate 3000 HPLC with variable wavelength detector, Thermo Scientific, Waltham, MA, USA), culture filtrates or ethyl acetate lipid extracts were taken up at 1 mg/mL concentration in 60% LC-MS grade water and 40% acetonitrile, supplemented with 0.1% formic acid (W6-4 Optima Water; A955-4, Acetonitrile; A117-50 Formic Acid; Fisher Scientific, Waltham, MA, USA). The column separation was performed on a ZORBAX Eclipse Plus C18, 95 Å, 4.6 × 250 mm, 5 µm, 400 bar pressure limit (cat. 959990-902, Agilent, Santa Clare, CA, USA) by applying an acetonitrile/water gradient at 1.2 mL/min flow rate, and a temperature of 60 °C. The mobile phase consisted of water, 0.1% formic acid (A), and acetonitrile 0.1% formic acid (B). The following gradient conditions were used: from 0 min at 5%, B ramped up to 40% B at 15 min, and ramped up to 90% B at 18 min, which was ramped down to 5% B at 23 min and kept at this concentration for 1 min post-run. Fractions were collected either at the corresponding variable wavelength detector peak for the pyochelin compound at 254 nm (proportional at 2 min retention time) or at the corresponding peak for peptides at 205 nm (proportional 4 min). The resulting fractions were evaporated to dryness under nitrogen to obtain purified extracts and re-diluted in the assay buffers with a stock concentration of 1 mg/mL and analyzed directly by LC-QTOF-MS/MS.

### 2.9. Compound Profiling by LC-QTOF-MS/MS Analysis

Samples obtained from culture filtrate fractionation, ethyl acetate extractions, or sample standards (i.e., pyochelin) were loaded at 0.1 µg/µL, with a 10 µL total load onto a 1290 Infinity II HPLC (Agilent, Santa Clare, CA, USA). A ZORBAX RRHD Eclipse Plus C18, 95Å, 2.1 × 50 mm, 1.8 µm, 1200 bar pressure limit (cat. 959757-902, Agilent, Santa Clare, CA, USA) was used for separation with a flow rate of 0.3 mL/min, and a temperature of 40 °C. The mobile phase consisted of water, 0.1% formic acid (A), and acetonitrile 0.1% formic acid (B). The following gradient conditions were used: at 0.5 min, 40% B ramped up to 95% B at 25 min, which was kept at this concentration for 5 min post-run. Following HPLC separation, the samples were analyzed in an Agilent 6546 QTOF mass analyzer (Agilent, Santa Clare, CA, USA) using a positive ion electrospray mode, acquisition range of 100–1700 atomic mass units, scan rate of 3 spectra/s, source at 225 °C with a gas flow of 12 L/min, sheath gas temperature of 300 °C, and sheath gas flow of 11 L/min. The VCap was set at 3500, the nozzle voltage at 1000 V, the fragmentor at 150, the skimmer at 65 V, and the octopole RF peak at 750. LC-QTOF-MS/MS fragmentation was completed at 20, 40, and 70 eV. Data acquisition was performed using MassHunter Data Acquisition Software (version 10.0, Agilent, Santa Clare, CA, USA). Data analysis was performed using the following tools: Agilent Profinder for raw file spectrum alignment; Mass Profiler Professional for statistical analysis, including identification of unique compounds by subtracting compounds present in the M9 minimum medium control; and personal compound database and library (PCDL) for compound identification with *Bacillus subtilis* and *Pseudomonas aeruginosa,* in addition to the METLIN 3.1.5 lipid library (G6825AA METLIN, Agilent, Santa Clare, CA, USA). MassHunter Qualitative Analysis Software was used to generate base peak chromatograms and compound mass-specific extracted ion chromatograms, including METLIN library alignment of compounds, by using the “Find by Formula” function for compound identification.

### 2.10. Fluorescence, Confocal and Scanning Electron Microscopy

Agar plug samples from selected microbial cultures were blotted onto glass slides, followed by sample lyophilization and gold coating using a sputter coater (DII Smart Coater, JEOL LTD., Peabody, MA, USA). Agar plug edges were analyzed using a scanning electron microscope (SEM; JCM-6000 PLUS Bench Top Scanning Electron Microscope, JEOL LTD., Peabody, MA, USA).

The live/dead^®^ BacLight^TM^ Bacterial Viability Kit (Molecular Probes, Invitrogen Waltham, MA, USA) was utilized to differentiate between live and dead cells, according to the manufacturer’s recommendations, with live cell dye SYTO 9 measured under green fluorescence and cell death dye PI measured under red fluorescence. For cell death determination of agar plug-contained cells and for cell death determination following Jet A fuel culture, samples were spotted onto glass slides. The samples were either analyzed by fluorescence microscopy, including phase-contrast settings, using a Nikon Eclipse Ti Fluorescence Microscope (Nikon Instruments Inc., Melville, NY, USA), or by confocal microscopy using a Nikon Ti2 C2 Eclipse Confocal Microscope (Nikon Instruments Inc., Melville, NY, USA) with a Nikon Eclipse Ti2 illumination system and a Nikon Eclipse Ti2 microscope camera under 10x/0.45 aperture (Plan Apo λ) (Control) or 20x/0.75 aperture (Plan Apo λ) (#133 cell-free filtrate) objectives, with excitation at 405 (blue, DAPI),—488 (green, GFP),—561 (red, TEXAS RED). The images were taken with NIS-Elements coupled to Denoise.ai software (Nikon Instruments Inc., Melville, NY, USA) to remove noise.

### 2.11. Flow Cytometry

Fuel microbial cultures were set up in 40 mL glass vials containing 5 mL water at the bottom (1× M9 minimal media), overlaid with 20 mL of filter-sterilized Jet A fuel as the carbon source. Fuel cultures were inoculated with Gram-positive bacterium *Gordonia* sp. at a final concentration of 1 × 10^5^ cells/mL. The cultures were supplemented with the chosen antimicrobials, e.g., crude culture filtrate, at doses of 0.0 µL (microbial growth control) and administered at 100 µL (proportional at 1.38 mg/mL compound mixture, derived from dry-weight determination), 500 µL, 1 mL, and 2.5 mL, followed by incubation at 28 °C under agitation. Samples were taken over the course of 7 days and 1 mL of each sample was collected across different time points. Serial dilutions were made for CFU culture plating to confirm the number of viable culturable cells. A negative control, with M9 + 0.5% glycerol and with 1 × 10^5^ cells/mL *Gordonia* sp., without any fuel, was sampled to confirm if only the filtrate was the reason for an antimicrobial effect and not the fuel itself. In addition, the cells were stained with live/dead^®^ BacLight^TM^ Bacterial Viability Kit (Molecular Probes, Invitrogen Waltham, MA, USA). SYTO 9 live cell dye was used at a concentration of 0.5 µM (green fluorescence) and cell death dye PI (red fluorescence) was used at a concentration of 0.25 µM. Flow cytometry (FCM) was performed using an Attune^TM^ N×T Flow Cytometer (Thermo Scientific, Waltham, MA, USA), including instrument performance testing using Attune^TM^ Performance Tracking beads (cat# 4449754, Thermo Scientific, Waltham, MA, USA). FCM analysis included the determination of live, dead, and total cell numbers. To exclude background signals, the fluorescence detection thresholds were set for green (488 nm/500 emission for SYTO 9 detection) and yellow (561 nm excitation/615 nm emission for PI detection) by gating on dot plots. Adjustment of forward-scatter was used for cell size and fluorophore detection. A side-scatter adjustment was used for the determination of cellular complexity to distinguish between cells and cellular debris. The percent of live cells, dead cells, lysed cells, and viable but non-culturable (VBNC) cells were determined by the following formulas:

Number of dead cells: PI positive red cells.
Number of live cells: PI-excluded Syto-9-stained green cells.
Total number of cells = number of live cells + number of dead cells.
Percent live cells = (number of live cells/total number of cells) × 100.
Percent dead cells = (number of dead cells/total number of cells) × 100.
Percent CFU = (CFU after exposure of antimicrobials/cells inoculated at the beginning of the experiment) × 100.
Percent VBNC cells = percent live cells—percent CFU.
Percent lysed cells = (cells inoculated at the beginning of the experiment—total number of cells)/cells inoculated at the beginning of the experiments) × 100.

### 2.12. Statistical Analysis

Statistical analysis for growth experiments included consideration of a number of independent samples (*n*), which included the average of at least triplicates of CFU plating. Two-way ANOVA was performed for multiple dose comparisons, with Dunnett’s post-test, using GraphPad Prism 10.0 software (Dotmatics, Boston, MA, USA).

## 3. Results

### 3.1. Agar Plug Biocontrol Agent Diffusion Screening

**Figure 1:**
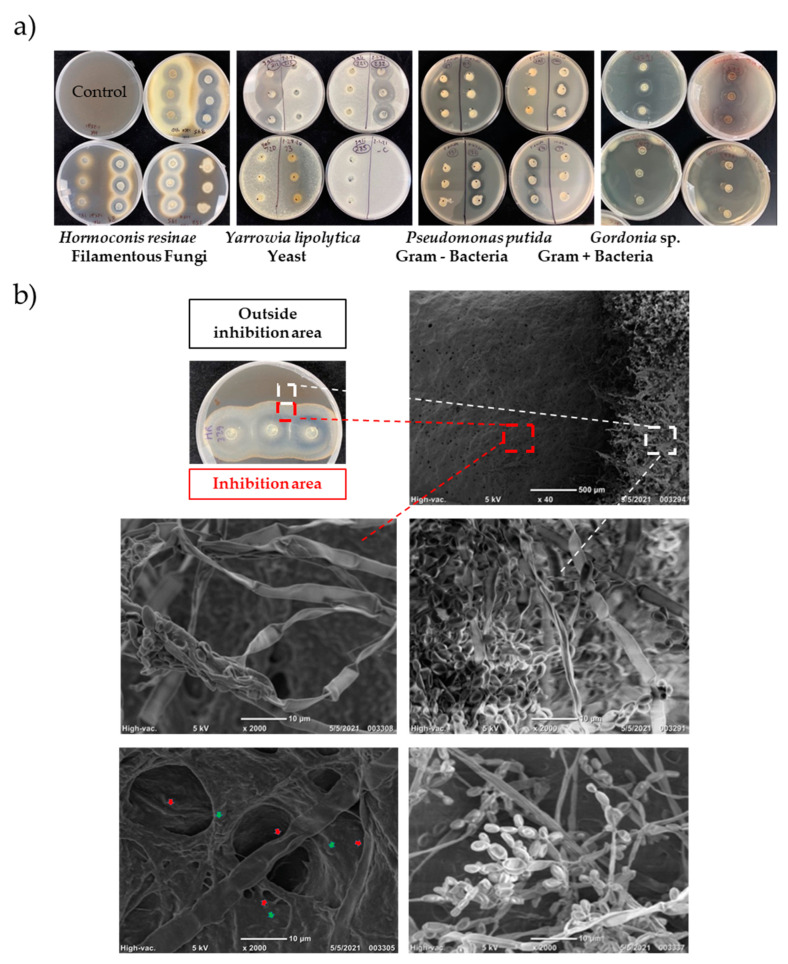
Agar plug screening of antimicrobial agent-producing microbes. (**a**) Agar plug screening of fuel isolates repository with the selected target microbes *P. putida*, *Gordonia* sp., *Y. lipolytica*, and *H. resinae*. (**b**) SEM images show *Hormoconis resinae* fungal mycelium and spores. The images show shrinking fungal mycelium and spores from the inhibited fungal growth area (red dashed square) compared to the intact fungal mycelium and spores from outside the inhibition area (white dashed square). The red arrows point to ultrastructure damages in both the fungal mycelium and spores while green arrows point to biocontrol *Delftia* sp. #329 bacteria.

Agar plug screening included a total of 496 microbes from our fuel isolate repository, which were tested for potential production of antimicrobials against four target microbial groups, including the Gram-negative bacteria *Pseudomonas putida*, Gram-positive bacteria *Gordonia* sp., yeast *Yarrowia lipolytica*, and filamentous fungi *Hormoconis resinae* (Table 1 and Figure 1a). These four microbes were selected because they are common aviation fuel contaminants and grow very well in fuel. The agar plug screening assay is based on donor biocontrol isolate agar plugs that are inserted into the chosen microbial test recipient culture plates, followed by measurement of microbial inhibition zones that form when active antimicrobial compounds diffuse into the adjacent plate area.

To distinguish cell-to-cell interactions’ possible false-positive results from the actual desired diffusion of secreted soluble antimicrobial compounds, inhibition zones were further analyzed by light/fluorescence microscopy and SEM. While light microscopy and fluorescence microscopy lacked the resolution to identify potential damage of fungal mycelia and spores (data not shown), SEM was able to detect damage resulting from cell-to-cell interactions. Using this method, we excluded some of the promising isolate candidates, including *Delftia* sp. #329. In detail, isolate #329 showed agar plug inhibition of *H*. *resinae*, but when tested by SEM, damage in hyphae and spore structures indicated cell-to-cell interactions, rather than damage due to antimicrobial compound diffusion (Figure 1b). In detail, the fungal mycelium localized inside the inhibition area was surrounded by biocontrol bacterial cells that seemed to induce fungal death (live/dead cells’ stain was positive, red) by the proximity of the bacteria to the fungal hyphae. This is also supported by detailed SEM images (Figure 1b) that show fungal mycelium and spores from the inhibited fungal growth zone compared to outside of the inhibition area.

Agar plug screening of the two isolates, *Pseudomonas protegens* #133 and *Bacillus subtilis* #232, demonstrated the highest antimicrobial activity against the four initially chosen test microbes *P. putida*, *Gordonia* sp., *Y. lipolytica*, and *H. resinae*. In addition, fluorescence and SEM microscopy analysis showed no visible evidence of cell-to-cell interactions. Therefore, these microbes were further tested against another group of fuel-contaminants, i.e., Gram-negative bacteria: *Acinetobacter venetianus*, *Hydrocarboniphaga effusa,* and *Escherichia coli* Jm109; Gram-positive bacteria: *Bacillus atrophaeus*, *Nocardioides albus*, and *Rhodococcus equi*; yeasts: *Candida ethanolica* and *Meyerozyma guilliermondii*; and filamentous fungi: *Aspergillus versicolor* and *Fusarium oxysporum*.

As shown in (Table 1), there is a consistency of biocontrol efficacy across the four different microbial classifications reflecting a wide range of applications for biocontrol products. It is noticeable that the tested strains of filamentous fungi showed the largest inhibition zones, followed by the Gram-negative bacteria, Gram-positive bacteria, and yeast as the most resistant among the four microbial classifications. It is remarkable that *P. protegens* #133 was more efficient against bacterial targets, whereas *B. subtilis* #232, with few exceptions, was more efficient against fungal targets.

### 3.2. Antimicrobial Activity Screening of Crude Filtrates from Bacillus subtilis #232 and Pseudomonas protegens #133 in Liquid Culture

In accordance with observations that a positive inhibition zone in agar plug screening can also result from biocontrol-cell-to-target-cell interactions and not necessarily from secreted antimicrobial agents, a liquid culture screening assay was developed using 96-well plates. The assay is based on the growing microbial hydrocarbon fuel contaminants in the presence of #133 or #232 fuel isolate culture filtrates to ensure that the testing is entirely limited to secreted soluble biocontrol compounds. Using the 96-well liquid culture assay, the chosen biocontrol isolates #232 and #133 were tested for antimicrobial activity against Gram-negative and Gram-positive bacteria, yeast, and filamentous fungi.

**Figure 2:**
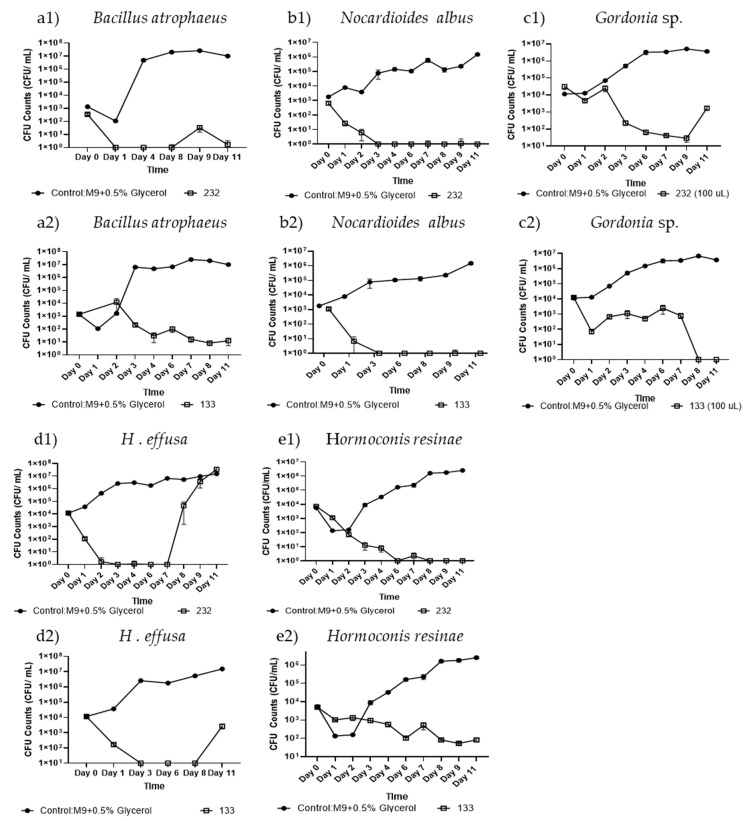
Antimicrobial activity screening of isolate *B. subtilis* #232 and *P. protegens* #133 crude culture filtrate in liquid culture. Antimicrobial activity of the crude culture filtrates from isolate *B. subtilis* #232 (**1**) and *P. protegens* #133 (**2**) was tested against (**a**) *B. atrophaeus*, (**b**) *N. albus*, (**c**) *Gordonia* sp., (**d**) *H. effusa*, and (**e**) *H. resinae* over 11 days, using CFU evaluation. The control shown in circles, and the test samples are in squares. A two-sample Student’s *t*-test was applied by taking the averages of each condition and combining all the control data across experiments. Error bars represent the standard error, with a sample size of *n* = 6 and above.

Isolate #232 crude culture filtrate testing over 11 days demonstrated complete growth inhibition of all tested Gram-positive bacteria, including *B. atrophaeus* (−6.78 log-fold change, *p* < 0.0005, *n* = 6, Figure 2(a1), *N. albus* (−6.17 log-fold change, *p* < 0.0001, *n* = 12, Figure 2(b1)), and *Gordonia* sp. (−3.34 log-fold change, *p* < 0.05, *n* = 12, Figure 2(c1)). In contrast, isolate #232 crude culture filtrate had limited antimicrobial activity against Gram-negative bacteria *A. venetianus*. However, #232 culture filtrate showed a −3.16 log-fold change, (*p* < 0.001, *n* = 6) against *E. coli* Jm109 (data not shown). While #232 culture filtrate yielded an initial growth reduction against *H. effusa* from day 1 to 7, the biocontrol activity seemed to be lost after day 8 (Figure 2(d1)). Interestingly, isolate #232 culture filtrate demonstrated effective antimicrobial activity for the fungal model organism *H. resinae*, (Figure 2(e1)) with an observed −6.58 log-fold change (*p* < 0.001, *n* = 18) compared to the control. However, culture filtrate from #232 had no antimicrobial activity against the yeast *Y. lipolytica* (data not shown).

Biocontrol activity testing of isolate #133 crude culture filtrate resulted in complete growth inhibition of all tested Gram-positive bacteria, including *B. atrophaeus* (Figure 2(a2)) (−5.91 log reduction, *p* < 0.0001, *n* = 12), *N. albus* (Figure 2(b2)) (−6.17 log-fold change, *p* < 0.0005, *n* = 6), and *Gordonia* sp. (Figure 2(c2)) (−6.57 log-fold change, *p* < 0.001, *n* = 9) by day 11. Similarly to #232, isolate #133 culture filtrate lacked biocontrol activity against the Gram-negative bacteria *E. coli* Jm109 cells (data not shown), while a reduced growth of *H. effusa* was observed by day 11, with a −3.75 log-fold change (*p* < 0.0001, *n* = 12) (Figure 2(d2)). In contrast, #133 culture filtrate was highly efficient at killing the filamentous fungus *H. resinae* (Figure 2(e2)) with a −4.48 log-fold change (*p* < 0.0005, *n* = 6) by day 11. However, #133 crude filtrate was not capable of inhibiting other filamentous fungi, e.g., *F. oxysporum* (data not shown).

### 3.3. Antimicrobial Activity Screening of Bacillus subtilis #232 and Pseudomonas protegens #133 Purified Compounds in Liquid Culture

Our selection standards for the ideal antimicrobial compounds contained within #133 and #232 culture filtrates included semi-polar molecules with good stability in the fuel phase to allow compound diffusion in water, biofilm, and hydrocarbon fuel phase without producing fuel emulsification. To better characterize compounds that match these criteria, two different culture filtrate purification methods were used, including ethyl acetate liquid/liquid extraction for the purification of semi-polar compounds, such as lipids and lipopeptides, and semi-preparative HPLC for the purification of lipopeptide compounds. The nitrogen-dried products from the purification steps were reconstituted prior to testing in a liquid screening assay. Aliquots of the liquid cultures were taken over a time course of 11 days and plated for CFU counting to assess compound antimicrobial activity.

**Figure 3:**
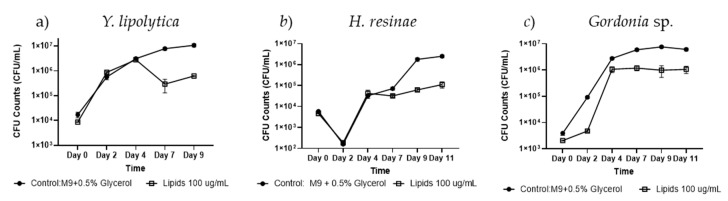
Antimicrobial activity screening of isolate *B. subtilis* #232 lipid extracts in liquid culture. Antimicrobial activity of isolate #232 lipid extracts at a concentration of 100 µg/mL was tested on (**a**) *Y. lipolytica*, (**b**) *H. resinae*, and (**c**) *Gordonia* sp. for 11 days using CFU evaluation. A two-sample Student’s *t*-test was applied by taking the averages of each condition and combining all the control data across experiments. Error bars represent the standard error, with a sample size of *n* = 3.

The testing of isolate #232 ethyl acetate-derived lipid extracts in 96-well liquid assay resulted in significant growth inhibition of the yeast *Y. lipolytica* at 100 µg/mL on day 9 with a −1.23 log-fold change (*p* < 0.05, *n* = 3, Figure 3a). Similarly, the growth of the filamentous fungus *H. resinae* did not drop below the 1 x log scale of the control on days 2–7 but a slight growth inhibition was observed on days 9–11 with a −1.35 log-fold change on day 11 (*p* = 0.08, *n* = 3, Figure 3b). In contrast, #232 lipid extracts seemed to only partially reduce the growth of *Gordonia* sp. with a −6.57 log-fold change (*p* < 0.01, *n* = 3, Figure 3c), yet lacked complete growth inhibition.

To identify uniquely produced antimicrobial compounds in the #232 filtrates, ethyl acetate liquid/liquid extractions were further analyzed by LC-QTOF-MS/MS at a concentration of 1 mg/mL. Of the 955 unique compounds discovered by LC-QTOF-MS/MS, library alignment with the *Bacillus subtilis* PCDL identified gageostatin C, with retention times (RT) at 16.305 and 18.806 min, and gageopeptin B (RT at 18.559 and 19.677 min) as the most abundant compounds with a library match identification score of 98.845 and 98.8, respectively (Supplemental Figures S1 and S2 and Data S1). In addition, ethyl acetate extracts derived from #232 culture filtrate also demonstrated a high abundance of molecules from the macrolactin family, including macrolactin B, macrolactin W, macrolactin F, 7-O-malonyl macrolactin, and 7-O-succinylmacrolactin that were distributed over retention times between 1.385 and 3.522. Repetition of filtrate purification and analysis of different #232 culture filtrate batches showed a high compound variability for macrolactins and other known antimicrobial compounds, i.e., bacillaene, chloretetain, and gageostastin, which caused difficulties in targeted single compound purification (data not shown). Therefore, the remainder of this study was focused on the #133 *P. protegens* strain.

**Figure 4:**
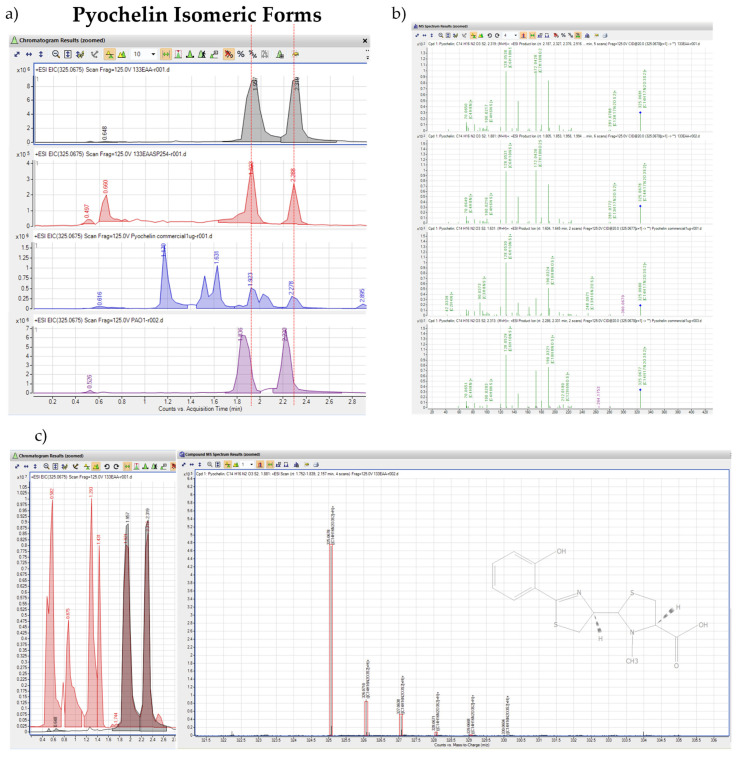
LC-QTOF-MS/MS analysis of isolate #133 culture filtrate containing compounds purified by ethyl acetate liquid extraction and semi-preparative HPLC fractionation. (**a**) Comparison of LC-QTOF-MS/MS mass spectrometry analysis on extracted ion chromatograms (EICs) of #133 culture filtrate ethyl acetate extracts (133EAA, grey trace), targeted pyochelin compound purification using semi-preparative HPLC collection of 254 nm trace (133 EAA SP 254, red trace), commercial pyochelin standard (blue trace), and ethyl acetate extracts from *P. aeruginosa* PAO1 culture filtrate (PAO1, purple trace) with known production of pyochelin. EICs for the pyochelin compound at a mass of 325.0625 Da are shown with the isomeric form of pyochelin indicated by red lines at an RT of approximately 1.89 and 2.3 min. (**b**) LC-QTOF-MS/MS-derived fragment ion spectrum of both prominent (325.0678 Da) pyochelin precursor ion peaks starting at 1.6 min and 2.1 min RT for #133 culture filtrate ethyl acetate extracts (133 EAA) in the two top panels compared to the commercial pyochelin standard (pyochelin commercial 1 µg, bottom panels). Different product ions and predicted formulas are in green, precursor ions are labeled with a blue square on top, and non-product ions are in purple. (**c**) Base peak chromatogram (BPC) for #133 culture filtrate ethyl acetate extracts (133 EAA, red trace) overlaid with two EICs extracted isomeric peaks for pyochelin (grey trace) with molecular ion spectrum, including compound formulas, different isotopes, and library isotopes matches in the red boxes.

Mass spectrometry analysis of #133 culture filtrate ethyl acetate extracts by LC-QTOF-MS/MS detected over 177 unique compounds, including common molecules contained in the biofilm of other *Pseudomonas* sp., such as quorum-sensors N-3-Oxo-Dodecanoyl-L-Homoserine lactone (3-oxo-C12-HSL) and Nonyl-4-Hydroxyquiniline-N-oxide (NQNO), and rhamnolipids Rha-C10:1-C8, Rha-C10-C10-CH3, Rha-Rha-C12-C10, Rha-C12:1-C10, and Rha Rha-Rha-C14-C14 (Supplemental Data S2). Interestingly, the compound trace for the siderophore pyochelin that is also produced in other *Pseudomonas* species, such as *P. aeruginosa* [34], was highly abundant in the ethyl acetate extracts. In detail, ion extraction for pyochelin at 325.0675 Da, [M+H+], resulted in dual peaks, representing potential isomeric forms at approximate retention times of 1.89 and 2.3 min (Figure 4a). The extracted ion chromatogram (EIC) for #133 pyochelin (Figure 4a, grey trace) matched the retention times of the extracted ion peaks (Figure 4a, blue trace) for the commercially purchased pyochelin I/II standard (Santa Cruz Biotechnology, Santa Cruz, CA, USA) and the extracted ion trace for pyochelin of *P. aeruginosa* strain PAO1 **(**Figure 4a, purple trace). Yet, commercial pyochelin (designated as synthetic, 96% pure, and NMR tested) seemed to have some visible impurities between retention times of 0.2 and 1.6 min.

The corresponding compound impurities in commercial pyochelin versus ethyl acetate extracted pyochelin were also visible in the corresponding MS/MS fragmentation spectra (Figure 4b) of the primary pyochelin ion at a mass of 325.0678 Da when looking at ions from both the 1.8 and 2.3 min RT peaks. While there was excellent overlap in fragment ions, there seemed to be a higher appearance of ion contaminants in the commercial pyochelin sample. When overlaying the #133 ethyl acetate extract’s (133 EAA) base peak chromatogram (BPC, Figure 4c, red trace) with the EIC trace for pyochelin (Figure 4c, grey trace), there is an absolute match for the two peaks, providing a potential easy cleanup due to the lack of other contaminating compounds at that retention time. The MS1 ion spectrum for the #133 pyochelin compound (Figure 4c) indicates excellent matches with the predicted ion isotopes with existing H+, Na+, and K+ adducts. To confirm the potential antimicrobial action of #133-produced pyochelin, ethyl acetate extracts were further purified by collecting fractions at the expected 254 nm UV absorbance trace for pyochelin, using a semi-preparative HPLC with a variable wavelength detector (Figure 4a, red trace). Following LC-QTOF-MS/MS analysis for quality control testing of pyochelin, the extracted compound fractions were used for testing in a 96-well plate antimicrobial activity assay.

**Figure 5:**
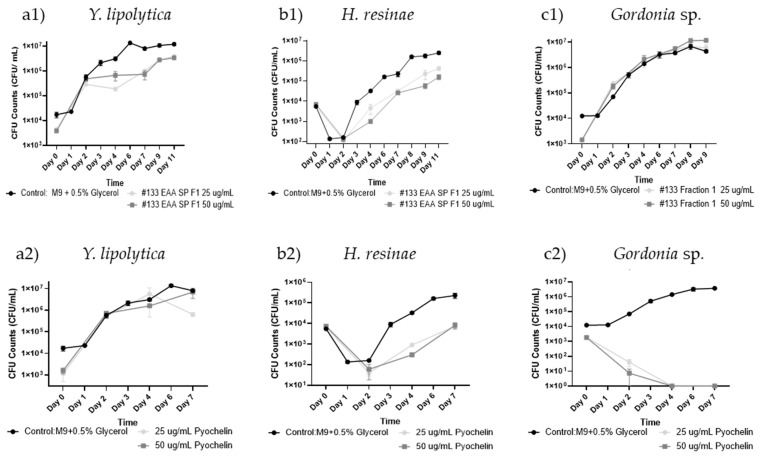
Antimicrobial activity screening of *P. protegens* #133 pyochelin extract in liquid culture. Antimicrobial activity of Isolate #133 pyochelin extracts (**1**) and commercial pyochelin standard (**2**) at concentrations of 25 and 50 µg/mL were tested on (**a**) *Y. lipolytica*, (**b**) *H. resinae*, and (**c**) *Gordonia* sp. over 11 days using CFU evaluation. A two-sample Student’s *t*-test was applied by taking the averages of each condition and combining all the control data across experiments. Error bars represent the standard error, with a sample size of *n* = 3.

When testing the antimicrobial activity of #133 purified pyochelin in 96-well plates, there was a slight growth inhibitory effect on the yeast *Y. lipolytica* and the filamentous fungus *H. resinae* with a −1.21 log reduction (*n* = 3) in the presence of 25 µg/mL at day 4, and a −1.51 log reduction (*n* = 3) in the presence of 50 µg/mL, respectively, compared to the controls (Figure 5(a1,b1)). In contrast, no antibacterial activity for #133 purified pyochelin was observed against the test microbe *Gordonia* sp. (Figure 5(c1)). In comparison, the commercial pyochelin standard was not effective for the growth inhibition of *Y. lipolytica* at the tested doses of 25 µg/mL and 50 µg/mL (Figure 5(a2)) while it exhibited a slight fungistatic effect against *H. resinae* with log-fold changes of −2.04 in the presence of 25 µg/mL (*p* < 0.04, *n* = 3) and −1.56 at 50 µg/mL (*p* < 0.01, *n* = 3) at day 4, respectively. Yet, at day 7, the inhibitory effect was limited to a log-fold change of −1.45 at a dose of 25 µg/mL (*p* < 0.052, *n* = 3) and −1.50 (*p* < 0.055, *n* = 3) at 50 µg/mL (Figure 5(b2))**.** Interestingly, commercial pyochelin yielded a significant growth inhibition of *Gordonia* sp. at both doses starting at day 2 with a log-change of −3.24 at 25 µg/mL (*p* < 0.05, *n* = 3) and a log-change of −4.02 (*p* < 0.05 l) at 50 µg/mL, compared to the control. By day 7, there was complete growth inhibition with a log-fold change of −6.57 (*p* < 0.04 l, *n* = 3) for both concentrations compared to the control (Figure 5(c2)).

The liquid screening assay results from microbial isolates *P. protegens* #133, and *B. subtilis* #232 crude culture filtrates, fractions, and commercial pyochelin are summarized in Table 2 below. The corresponding growth inhibition results representing biocidal activity are displayed.

### 3.4. Antimicrobial Activity of P. protegens #133 Crude Culture Filtrate and Pure Pyochelin in Jet A Fuel Microbial Consortium Culture

To test the suitability of #133 crude culture filtrate and pyochelin as biocontrol agents in hydrocarbon environments, Jet A fuel consortium culture assays were established. The microbial consortia consisted of the bacteria *P. putida* and *Gordonia* sp., the yeast *Y. lipolytica*, and the filamentous fungi *H. resinae,* inoculated at a total final concentration of 1 × 10^4^ CFU/mL in 5 mL minimal medium (1:1 mixture of M9/YNB) overlaid with 20 mL of Jet A fuel (fuel phase). For antimicrobial activity testing, cultures were supplemented with antimicrobial isolate #133 *P. protegens* crude culture filtrate at 100 µL (representing approximately 1.38 mg of the compound mixture dry weight), 1 mL, or 2.5 mL total volume or pyochelin standard at 20 µg/mL.

**Figure 6:**
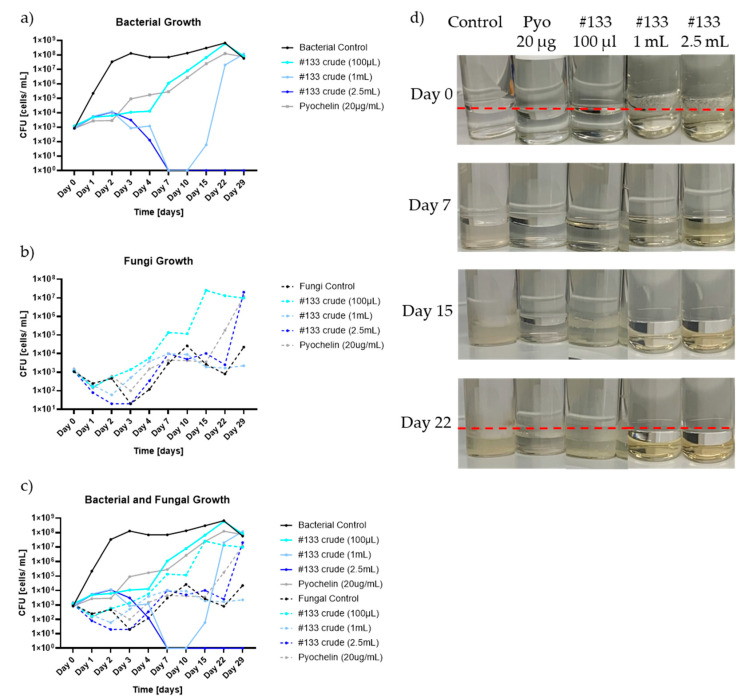
Microbial consortium growth inhibition dynamics after treatment with isolate *P. protegens* #133 crude culture filtrate in fuel culture. Antimicrobial activity of #133 crude culture filtrate (#133 crude) and pyochelin administered at different doses with fuel consortium cultures containing *P. putida*, *Gordonia* sp., *Y. lipolytica*, and *H. resinae*. The graphs display CFU/mL determined for (**a**) total bacteria (*P. putida* and *Gordonia* sp.); (**b**) total fungi (*Y. lipolytica* and *H. resinae*); and (**c**) total microbial load (bacteria plus fungi), across different time points (day 0 to day 29). Testing included triplicate CFU plating of *n* = 1 sample each. (**d**) Consortium growth over the course of the experiment for 29 days. The control (inoculated microbial consortium without antimicrobial treatment) pyochelin of 20 µg/mL and isolate #133 crude culture filtrate supplemented at different doses of 100 µL, 1 mL, and 2.5 mL. The red line indicates the interphase between the fuel phase and the water bottom.

Antimicrobial testing using a dose of 100 µL of #133 crude culture filtrate had no visible effect on bacterial growth (Figure 6a). A dose increases of #133 filtrate to 1 mL resulted in a visible antibacterial effect until day 15 (a −6.36 log-fold reduction compared to the control), which was followed by a steep increase in bacterial growth until the end of the study. A further dose increases of #133 filtrate crude filtrate to 2.5 mL demonstrated a −7 log-fold reduction compared to the control, resulting in complete growth inhibition of bacteria at day 29 (Figure 6d). Pyochelin tested at 20 µg/mL showed a log-fold reduction of −2.39 on day 7, yet bacterial growth returned to control levels on day 29. In contrast, there was no fungal growth inhibition observed in the presence of either biocontrol, #133 crude culture filtrate, or pyochelin at the administered doses (Figure 6b). Figure 6c shows the bacterial and fungal growth graphs together from day 0 to 29.

### 3.5. Antimicrobial Cytotoxicity Effect of P. protegens #133 Culture Filtrate Against Gordonia sp. in Jet A Fuel Culture Using Live/Dead Flow Cytometry

According to the above observed antibacterial effects of isolate #133 crude culture filtrate in liquid and in Jet A fuel cultures, functional fluorescent stains and flow cytometry were used to determine the mechanism of action, i.e., cell wall damage and cell death. Since *Gordonia* sp. appeared to be the most sensitive microbe to the antimicrobial actions of #133 culture filtrate, a corresponding single bacteria fuel culture assay was set up under similar conditions as the previous fuel consortium culture. Following the collection of the water phase, after different time points, cells were stained with live/dead dyes SYTO 9/Propidium iodide (PI) and analyzed by flow cytometry (FCM). The FCM dye exclusion assay allows the detection of SYTO 9 dye uptake by viable cells that can be easily distinguished from PI-stained membrane-damaged dead cells. To differentiate between viable metabolically active and viable but non-culturable cells (VBNC), CFU plate counts were performed.

**Figure 7:**
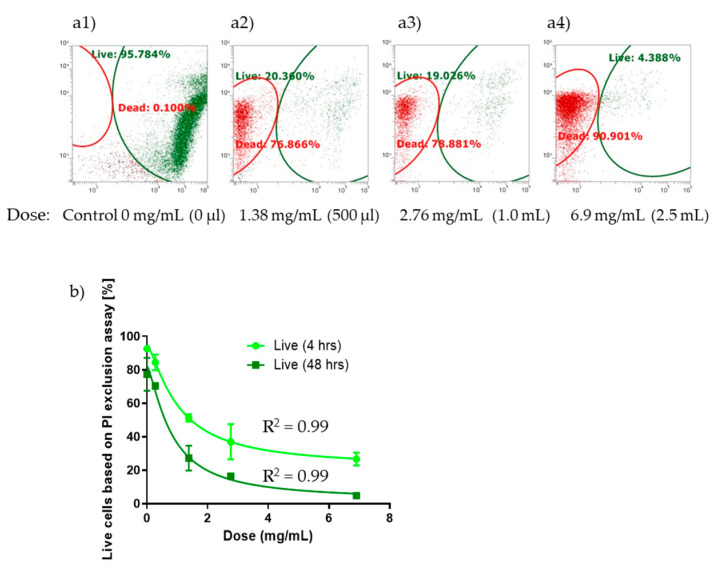
Antimicrobial cytotoxicity effect of *P. protegens* #133 culture filtrate against *Gordonia* sp. in Jet A fuel cultures using live/dead flow cytometry. *Gordonia* sp. growth in the presence of different doses of biocontrol #133 culture filtrate in Jet A fuel cultures determined by live/dead FCM over 7 days. The control (M9 minimal medium) and isolate #133 crude culture filtrate were tested at different concentrations of 100 µL (0.276 mg/mL), 500 µL (1.38 mg/mL), 1000 µL (2.76 mg/ mL), and 2500 µL (6.9 mg/mL). (**a**) Example of FCM live (SYTO 9, green)/dead (PI, red) cellular detection 48 h after exposure to #133 culture filtrate at different calculated doses considering a total volume of 5 mL as the aqueous bottom: (**a1**) Control, 0 mg/mL (0 µL), (**a2**) 1.38 mg/mL (500 µL), (**a3**) 2.76 mg/mL (100 µL), and (**a4**) 6.9 mg/mL (2500 µL). Green= live; Red= dead. (**b**) Cell viability dose–response curve after 4 h and 48 h of exposure to different doses of #133 culture filtrate with calculated concentrations given in (mg/mL). Error bars represent the standard error, with a sample size of *n* = 3.

FCM analysis using live/dead staining was used to assess the antimicrobial potency and efficacy under different doses of #133 culture filtrate, i.e., 100 µL (0.276 mg/mL), 500 µL, 1000 µL, and 2500 µL, by measuring relative green and red fluorescence of live and dead cells after 4 h and 48 h (Figure 7). According to the #133 culture filtrate dose–response determination using FCM live/dead analysis, the lowest dose of 100 µL culture filtrate had no effect on cell viability following 4 h and 48 h of exposure. In contrast, culture filtrate administration at increasing doses of 500 µL, 1 mL, and 2.5 mL resulted in reduced cell viability. At the lowest effective dose of 500 µL, the percent of viability was reduced to 51.26 ± 2.23% after 4 h while it dropped to 27.26 ± 3.81% after 48 h (*n* = 3). The highest reduction in percent of cell viability was observed in the presence of 2.5 mL of #133 culture filtrate with 26.69 ± 7.42% after 4 h, which dropped to 4.91 ± 0.23% after 48 h.

To determine the IC50 (the concentration of antimicrobial agent where the percent of inhibition is equal to 50 percent), a non-linear regression theoretical curve fit was applied. Non-linear regression analysis demonstrated good curve fits with an IC50 dose of 1.031 mg/mL (R^2^ = 0.9984) at the 4-h time point versus an even more effective IC50 dose of 0.8019 mg/mL (R^2^ = 0.9909) after 48 h. In addition, differences in the dose–response curve determined Hill slopes at 4 h (−1.352) to 48 h (−1.378), supporting the potency and efficacy of the #133 *P. protegens* biocontrol agent to effectively decrease *Gordonia* sp. cell viability.

On day 7, FCM could not detect a significant number of live or dead cells, with a low appearance of overall fluorescence intensity. Further evidence was given by FCM side-scatter adjustment that indicated visible changes in the light-scattering pattern typical for cellular debris from bacterial lysis containing ruptured cellular membranes. The overall lack of intact viable cells at day 7 was further confirmed by confocal microscopy (Supplemental Figure S3). Based on the day 7 FCM observation, we estimated that over 94% of the inoculated cells were lysed when exposed to the different #133 culture filtrate doses of 500 µL, 1 mL, and 2.5 mL (Table 3). While prolonged exposure to #133 culture filtrate resulted in FCM detection of SYTO 9 fluorescence-positive cells, no colony growth was observed following CFU culture plating. The FCM-detected viable cells are, therefore, depicted in Table 3 as VBNC. Besides the inactive growth behavior in the CFU culture plating, the VBNC demonstrated a #133 culture filtrate dose-dependent decrease in FCM detected cells with only 4.11% of viable cells remaining at 500 µL (*n* = 3), which was further lowered to 2.73% at 1 mL, and 1.08% at 2.5 mL of filtrate.

## 4. Discussion

This study aimed to identify novel naturally produced antimicrobial compounds for the control of biocontamination in hydrocarbon fuels. Initial antimicrobial activity testing of 496 microbial fuel isolates from our in-house repository by the commonly used agar plug screening method [17,35,36] resulted in several promising candidates. To exclude fuel isolates with false-positive antimicrobial activity resulting from cell-to-cell interactions, culture filtrate-contained antimicrobial soluble antimicrobial compounds were tested in specifically designed liquid culture assays. As a result, two fuel-isolate strains, *P. protegens* #133 and *B. subtilis* #232, demonstrated promising biocontrol activity against bacteria, yeast, and filamentous fungi.

### 4.1. Purification and Analysis of Antimicrobials from Bacillus subtills #232

Antimicrobials from *Bacillus* species are well-known and have been proposed for use in agricultural applications and clinical settings, including the production of biosurfactants [37,38,39,40]. According to the agar plug screening, isolate #232 had the largest inhibition zones against other Gram-positive bacteria *Gordonia* sp. and *B. atrophaeus*, Gram-negative bacterium *P. putida* and *H. effusa*, yeasts *Y. lipolytica*, *M. guilliermondii*, and *C. ethanolica*, and filamentous fungi *H. resinae*, *A. versicolor,* and *F. oxysporum*. Liquid culture assay of #232 culture filtrate demonstrated similar biocontrol efficiency for most of the above-tested microbes, especially the fungal targets, yet lacked the agar plug testing observed efficacy against the yeasts *Y. lipolytica*, *M. guilliermondii*, and *C. ethanolica*, and the filamentous fungi *A. versicolor* and *F. oxysporum*. This can have several reasons, including the stability of some of the compounds, and the lack of cell-to-cell interactions. For instance, some strains of *Bacillus* require cell-to-cell interaction for the activation of cellular envelope-localized stress-sensing receptors to initiate the production of different antimicrobial molecules. In addition, different *Bacillus* strains are known to produce volatile organic compounds with antifungal properties [23,26,27] that can be retained in the agar test plate but might evaporate during liquid testing.

According to the mass spectrometry analysis of *B. subtilis* #232 lipid extracts, one of the detected antifungal compounds, i.e., the hyphae and spore lysing antifungal lipopeptide fengycin C [41,42], was abundantly present, yet chemical instability of fengycins has been previously observed by other investigators [43]. Also, mass spectrometry detected the lipopeptide biosurfactant surfactin, which is known to cause osmotic pressure imbalance in fungi and bacteria [44,45]. When looking at potential active compounds that might be relevant for the observed inhibition of fungi and bacteria, gageostatin C was detected at high abundance [46] together with the zoospore motility inhibitor gageopeptin B [21]. Mass spectrometry also detected several abundant compounds of the macrolactin family, including 7-O-Malonyl macrolactin, macrolactin F, and macrolactin B, that have been demonstrated to exhibit antimicrobial activity against Gram-positive and Gram-negative bacteria [47,48]. Unfortunately, our efforts at targeted compound purification from #232 culture filtrate yielded variable results, although growth conditions and purification protocols were meticulously performed. The variable metabolic compound production of different *Bacillus* strains has been observed by other investigators [49,50] and was previously discussed as a result of “bacterial cannibalism” or cross-feeding, where a differentiated subpopulation harvests nutrients from their genetically identical siblings through metabolic exchange into metabolically distinct subpopulations.

### 4.2. Purification and Analysis of Antimicrobials from Pseudomonas protegens #133

The #133 *P. protegens* strain demonstrated high antimicrobial activity against Gram-positive and Gram-negative bacteria in agar plug testing, with large inhibition zones starting above 20 mm for most of the tested bacteria. In addition, #133 also demonstrated efficacy against all the tested fungi, with the radius of inhibition zones smaller than 20 mm. Compared to assays using the #232 isolate, which was efficient towards fungal targets, liquid culture assays of #133 lacked antimicrobial activity against the yeasts *Y. lipolytica*, *M. guilliermondii*, and *C. ethanolica*, and the filamentous fungi *A. versicolor* and *F. oxysporum*. Similar to *Bacillus* sp., *Pseudomonas* sp. produces VOCs [51] and phenolic compounds [52] with demonstrated antimicrobial activities that might be inactive in liquid culture assays. In contrast to *Bacillus* sp., most of the antimicrobial compounds produced by *P. protegens* include small molecules and lipid compounds, such as rhamnolipids and quorum-sensing molecules, including pyoverdine, pyocyanin, and phenzine [53]. Analysis of lipid extracts from #133 culture filtrate by mass spectrometry detected common molecules contained in the biofilm of other *Pseudomonads*, such as quorum-sensors 3-oxo-C12-HSL (N-3-Oxo-Dodecanoyl-L-Homoserine Lactone) and NQNO (Nonyl-4-Hydroxyquiniline-N-oxide), and rhamnolipids Rha-C10:1-C8, Rha-C10-C10-CH3, Rha-Rha-C12-C10, Rha-C12:1-C10, and Rha Rha-Rha-C14-C14. In addition, the siderophore pyochelin [28] was identified as one of the most abundant compounds in #133 lipid extracts.

The compound trace for #133 extracted pyochelin differed from that of *P. aeruginosa* PAO1 by displaying two unique peaks at retention times 1.89 and 2.3 min, with practically no surrounding contaminating compounds, while *P. aeruginosa* had a much lower pyochelin compound abundance, with several other surrounding compound ions present. While the pyochelin iron chelating and associated antimicrobial action through metal ion depletion has been well-characterized [28,34], pyochelin was also suggested to cause cell death by bacterial membrane disruption [54]. The #133 culture filtrate purified pyochelin demonstrated low-level fungistatic effects for *Y. lipolytica* and *H. resinae* but lacked antimicrobial efficiency against *Gordonia* sp. In contrast, the purchased synthetic pyochelin standard lacked inhibition of *Y. lipolytica* but showed a similar fungistatic effect against *H. resinae,* with a significant bactericidal effect on *Gordonia* sp. Potential issues with the #133 pyochelin compound purity versus additional contaminants within the synthesized pyochelin product at a marketed 96% purity might affect the dose determinations, explaining potential differences in compound activity.

The Jet A fuel microbial inhibition assay contained a microbial consortia of major fuel contaminants, including *Gordonia* sp., *P. putida*, *Y. lipolytica*, *and H. resinae,* which are often found in a fuel tank environment. In contrast to liquid culture assays, Jet A fuel culture testing of same-dose commercial pyochelin standard (20 µg/mL) lacked effective inhibition of bacterial growth. The observed effect was similar to the lowest #133 culture filtrate dose of 100 µL, a dose that was only bacteriostatic. Bacterial growth stalled for 10 days when the dose of #133 was increased to 1 mL, while growth was eliminated at 2.5 mL. In contrast, fungal growth demonstrated an upward trend in the presence of bacterial inhibition. This observation suggests that the presence of bacteria in the consortium inherently exerts a growth inhibitory effect over the fungi, and when this effect is eliminated, fungal growth is enhanced. The equilibrium between growth-inhibitory and stimulatory effects of bacterial and fungal consortia is a phenomenon commonly observed in various co-culture scenarios [55,56].

While the Jet A fuel consortium culture was set up to test #133 culture filtrate efficiently against Gram-positive and Gram-negative bacteria, our observations concluded that #133 culture filtrate is highly efficient in killing *Gordonia* sp. Several species of *Gordonia* have been identified in hydrocarbon fuels with the ability to survive and proliferate in the toxic fuel environment by forming micelles [57,58,59,60]. Accordingly, the identified rhamnolipid compounds Rha-C10:1-C8, Rha-C10-C10-CH3, Rha-Rha-C12-C10, Rha-C12:1-C10, and Rha Rha-Rha-C14-C14 that are known surfactants might interfere with potential antimicrobial activity of other compounds contained in #133 culture filtrate in fuel by assisting micelle formation [5,61,62]. Yet, small molecules, such as pyochelin and other compounds contained in #133 culture filtrate, might act in destabilizing the cell membrane of these Gram-positive bacteria [63]. The mass spectrometry analysis detected the quorum-sensing molecules 3-oxo-C12-HSL [64] and NQNO might further assist in inhibiting bacterial growth [65]. It remains to be determined which compound in #133 culture filtrate is responsible for the observed antimicrobial action against Gram-positive bacteria.

### 4.3. Antimicrobial Mechanism Action of P. protegens #133 Filtrate in Jet A Fuel Using Live/Dead Flow Cytometry

The determination of the antimicrobial mode of action is crucial for compound optimization and the development of suitable commercial biocides. By using FCM live/dead analysis in combination with CFU culture plating, a common method to assess viable, VBNC, dead, and lysed bacterial cells [31,32,33,66,67], we were able to determine the #133 culture filtrate antimicrobial mode of action on Gram-positive *Gordonia* sp. bacteria when grown in Jet A fuel cultures. The corresponding data from FCM live/dead analysis, together with confocal microscopy analysis, following 48 h and 7-day exposure, confirm that antimicrobial compounds contained in #133 culture filtrate exhibit a bactericidal effect against *Gordonia* sp. through membrane damage-dependent bacterial cell lysis. In addition, the FCM determined a decrease in the IC50 from 1.031 mg/mL at 4 h versus a measured IC50 of 0.8019 mg/mL after 48 h exposure, which also suggests that the #133 culture filtrate antimicrobial effect on *Gordonia* sp. cell viability is not only dose-dependent but also has a time-dependent component. The observed bactericidal effect of #133 culture filtrate is further supported by FCM, which detected a low number (1.08% to 4.11%) of remaining viable cells at 7 days that were non-culturable, supporting a halt of bacterial metabolism through the antimicrobial effect as a further cause of cytotoxic cell death [32]. While the effective dose of #133 culture filtrate is relatively high with an IC50 of 0.8 mg/mL, it needs to be considered that the culture filtrate is composed of a multitude of compounds that when purified might result in much lower IC50 for individual compounds. The biocontrol activity of #133 culture filtrate seems to be unique in its capability to lyse the thick peptidoglycan-containing bacterial cell wall [68] of *Gordonia* sp. and even shows efficacy in the presence of hydrocarbon-based Jet A fuel.

## 5. Conclusions

In this study, 496 microbial fuel isolates from our in-house repository were screened to identify new endogenously produced antimicrobial compounds. Using agar plug screening, liquid culture growth testing, and Jet A fuel culture assays, the two fuel-isolate strains, *Pseudomonas protegens* #133 and *Bacillus subtilis* #232, demonstrated promising biocontrol activity against bacteria, yeast, and filamentous fungi. The *B. subtills* #232 culture filtrate contained antimicrobial compounds that seemed to be more efficient against fungal targets. In contrast, liquid cultures and fuel consortium cultures revealed an antimicrobial efficiency of #133 culture filtrate against Gram-positive bacteria. This was further evident by FCM-based antimicrobial mode of action testing using Jet A fuel-based bacterial cultures, where #133 culture filtrate exhibited a bactericidal effect against *Gordonia* sp.. Potential purified compounds that exhibit the #133 bactericidal activity should be considered in combination with other biocidal agents for the sustainment of hydrocarbon-based aviation fuels.

## Figures and Tables

**Table 1:** Agar plug screening of selected high-efficiency biocontrol microbes *Pseudomonas protegens* #133 and *Bacillus subtilis* #232 against 14 target strains. The radius of inhibition zones in [mm]: the darker green color in the gradient indicates the largest inhibition zones observed in the presence of biocontrol isolates #133 or #232. Gram-negative bacteria included *P. putida*, *A. venetianus, H. effusa*, and *E. coli* Jm109. Gram-positive bacteria included *Gordonia* sp., *R. equi*, *N. albus*, and *B. atrophaeus*. Yeasts included *Y. lipolytica*, *M. guilliermondii*, and *C. ethanolica.* Filamentous fungi included *H. resinae*, *A. versicolor*, and *F. oxysporum*. ± stands for the standard deviation of the listed sample size *n*. Days are listed where the diffusion assay had the largest inhibition zone, which was different due to high variability in the growth behavior of the tested bacteria, yeast, and filamentous fungi.

Target Microbes	Classification	*Pseudomonas protegens #133* (Gram—Negative)			*Bacillus subtilis #232* (Gram—Positive)		
		Inhibition Zone Radius [mm]	Time [Days]	*n*=	Inhibition Zone Radius [mm]	Time [Days]	*n*=
*Pseudomonas putida*	Gram-negative	30.17 ± 5.11	6	6	7.75 ± 14.31	7	12
*Acinetobacter venetianus*	Gram-negative	20.61 ± 14.48	7	18	3.17 ± 3.99	1	18
*Hydrocarboniphaga effusa*	Gram-negative	32.50 ± 4.51	7	6	3.83 ± 1.72	4	6
*Escherichia coli* Jm109	Gram-negative	23.67 ± 2.31	3	3	0.00 ± 0	5	3
*Gordonia* sp.	Gram-positive	30.33 ± 3.88	6	6	7.50 ± 11.8	6	12
*Rhodococcus equi*	Gram-positive	28.00 ± 2.00	3	3	2.67 ± 0.58	7	3
*Nocardioides albus*	Gram-positive	32.00 ± 6.48	6	9	4.22 ± 3.46	6	9
*Bacillus atrophaeus*	Gram-positive	16.83 ± 6.70	2	6	4.17 ± 2.79	1	6
*Yarrowia lipolytica*	Yeast	9.17 ± 5.92	4	12	20.50 ± 8.62	5	6
*Meyerozyma guilliermondii*	Yeast	17.33 ± 1.53	5	3	5.17 ± 1.80	3	12
*Candida ethanolica*	Yeast	3.61 ± 3.5	1	18	11.67 ± 5.16	3	18
*Hormoconis resinae*	Filamentous fungus	13.17 ± 5.14	4	24	26.33 ± 3.05	6	3
*Aspergillus versicolor*	Filamentous fungus	10.89 ± 3.23	3	18	20.33 ± 9.23	2	21
*Fusarium oxysporum*	Filamentous fungus	11.56 ± 4.85	2	18	19.71 ± 4.29	2	21

**Table 2:** Summary table of liquid culture antimicrobial activity testing. Antimicrobial activity of crude culture filtrates #133 and #232, purified HPLC fractions #133 EAA SP F2 (#133 ethyl acetate extractions, then semi-prep Fraction 2) and #232 SP F1 (#232 semi-prep Fraction 1), and commercial pyochelin. Calculated concentrations from total volume of culture are given in mg/mL or µg/mL. Log-fold change on day 11, bold• represents day 9, and underlined represents day 7, as not all testing ended on day 11. Representative *p* values are displayed as* *p* < 0.05, ** *p* < 0.001 and *** *p* < 0.0001.

Classification	Microbe	Growth Effect		Log-Fold Change on Day 11 (Efficient Growth Inhibition in Blue) (Bold • is Day 9 and Underline is Day 7)				
		133 Crude (6.9 mg/mL)	232 Crude (9.8 mg/mL)	133 Crude (6.9 mg/mL)	232 Crude (9.8 mg/mL)	Commercial Pyochelin (50 µg/mL)	133 EAA SP F1 (100 µg/mL)	232 SP F2 (100 µg/mL)
Gram-positive	*Gordonia* sp.	Kills	Kills	−6.57 **	−3.34 *	−6.57 **	**0.07** •	−0.05
Gram-positive	*Rhodococcus equi*	Reduces	No Effect	−2.09	0.95	not tested	not tested	not tested
Gram-positive	*Nocardioides* *albus*	Kills	Kills	−6.17 **	−6.17 ***	not tested	not tested	**0.33** •
Gram-positive	*Bacillus atrophaeus*	Kills	Kills	−5.91 ***	−6.78 **	not tested	not tested	not tested
Gram-negative	*Pseudomonas putida*	Enhances	No Effect	0.39	−0.25	not tested	not tested	not tested
Gram-negative	*Acinetobacter* *venetianus*	No Effect	Reduces	0.13	−1.02	not tested	not tested	not tested
Gram-negative	*Hydrocarboniphaga* *effusa*	Kills	No Effect	−3.75 ***	0.36	not tested	not tested	not tested
Gram-negative	*Escherichia coli* Jm109	No Effect	Reduces	−0.54	−3.16 **	not tested	not tested	not tested
Filamentous fungus	*Hormoconis* *resinae*	Kills	Kills	−4.48 **	−6.58 **	−1.5	−0.77	−0.84
Filamentous fungus	*Aspergillus* *versicolor*	No Effect	No Effect	**−1.93** •	−1.52	not tested	not tested	not tested
Filamentous fungus	*Fusarium* *oxysporum*	No Effect	No Effect	−0.42	−0.05	not tested	not tested	not tested
Yeast	*Meyerozyma guilliermondii*	No Effect	No Effect	0.14	0.03	not tested	not tested	not tested
Yeast	*Candida* *ethanolica*	No Effect	Enhances	**−0.32** •	2.48	not tested	not tested	not tested
Yeast	*Yarrowia* *lipolytica*	No Effect	Reduces	−0.47	−0.9	−1.09	−0.48	−0.35
Yeast	*Candida* *albicans*	No Effect	No Effect	−0.85	1.2	not tested	not tested	not tested

**Table 3:** The day 7 antimicrobial cytotoxicity effect of *P. protegens* #133 culture filtrate against *Gordonia* sp. in Jet A Fuel cultures using live/dead flow cytometry. *Gordonia* sp. growth in the presence of different doses of biocontrol #133 culture filtrate in Jet A fuel cultures determined by live/dead FCM over 7 days. The control (M9 minimal medium) and isolate #133 crude culture filtrate were tested at different doses of 500 µL, 1000 µL, and 2500 µL, with calculated doses of culture filtrate given in mg/mL considering a total volume of 5 mL water bottom. The table displays CFU plate counts, percent of dead Propidium iodide-stained cells, lysed cells (side-scatter determination), and SYTO 9-stained cells percent of viable but non-culturable cells (VBNC). The percentages were calculated based on the day 0 inoculum. Error is given as the standard error, with a sample size of *n* = 3.

Dose #133 Culture Filtrate	% CFUs	% Dead (PI-Stained Cells)	% Dead (Lysed Cells)	% VBNC (STYO9-Stained Cells)	Total Percent
500 µL (1.38 mg/mL)	0	0.99 ± 0.27	94.90 ± 1.24	4.11 ± 0.97	100.00
1000 µL (2.76 mg/mL)	0	1.12 ± 0.18	96.15 ± 1.14	2.73 ± 0.98	100.00
2500 µL (6.9 mg/mL)	0	2.12 ± 0.35	96.80 ± 0.31	1.08 ± 0.12	100.00

## Data Availability

The original contributions presented in this study are included in the article/Supplementary Material. Further inquiries can be directed to the corresponding authors.

## Supplementary Materials

The following supporting information can be downloaded at: https://www.mdpi.com/article/10.3390/biom15020227/s1, Supplemental Data S0. A list of all 496 microbial fuel isolates tested with agar plug screenings against *Pseudomonas putida* and the top 89, top 18, and top 9 isolates are displayed. Supplemental Figure S1: isolate #232 culture filtrate containing compounds purified by ethyl acetate liquid extraction, analyzed by LC-QTOF-MS/MS; Supplemental Figure S2: *Bacillus subtilis* specific compounds identified by LC-QTOF-MS/MS in fuel isolate #232; Supplemental Data S1: isolate #232 culture filtrate containing compounds purified by ethyl acetate extraction, analyzed by LC-QTOF-MS/MS; Supplemental Data S2: isolate #133 culture filtrate containing compounds purified by ethyl acetate extraction compared to lipid extracts from *Pseudomonas aeruginosa* PAO1 and commercial pyochelin, analyzed by LC-QTOF-MS/MS; Supplemental Figure S3: antimicrobial effect of #133 culture filtrate on *Gordonia* sp. in Jet A fuel cultures.

## Distribution Statement A

Approved for Public Release; Distribution is Unlimited. CLEARED on 22 January 2025; PA# AFRL-2025-0324).

## Abbreviations

AMPs	Antimicrobial Peptides
BPCs	Base Peak Chromatograms
CFU	Colony Forming Unit
EIC	Extracted Ion Chromatogram
FCM	Flow Cytometry
HPLC	High-Performance Liquid Chromatography
QTOF	Quadrupole Time-of-Flight
MS/MS	Tandem Mass Spectrometry
MIC	Minimum Inhibitory Concentration
M9	M9 Culture Medium
PBS	Phosphate Buffered Saline
PDA	Potato Dextrose Agar
PDB	Potato Dextrose Broth
PI	Propidium Iodide
PCDL	Personal Compound Database and Library
RPM	Revolutions Per Minute
RT	Retention Time
SMAP	Sheep Myeloid Antimicrobial Peptide
SEM	Scanning Electron Microscopy
TSA	Tryptic Soy Agar
TSB	Tryptic Soy Broth
VBNC	Viable But Non-Culturable
YNBs	Yeast Nitrogen Base without Amino Acids
VOCs	Volatile Organic Compounds
