# Supplementary material for: Identification of Pseudomonas protegens and Bacillus subtilis Antimicrobials for Mitigation of Fuel Biocontamination
Source: Biomolecules. 2025 Feb 4;15(2):227. doi: 10.3390/biom15020227 (PMC11853459; doi:10.3390/biom15020227)
Supplement: Supplementary file 1 [file biomolecules-15-00227-s001.zip › 20250122SupplementalFigure S1.pdf]

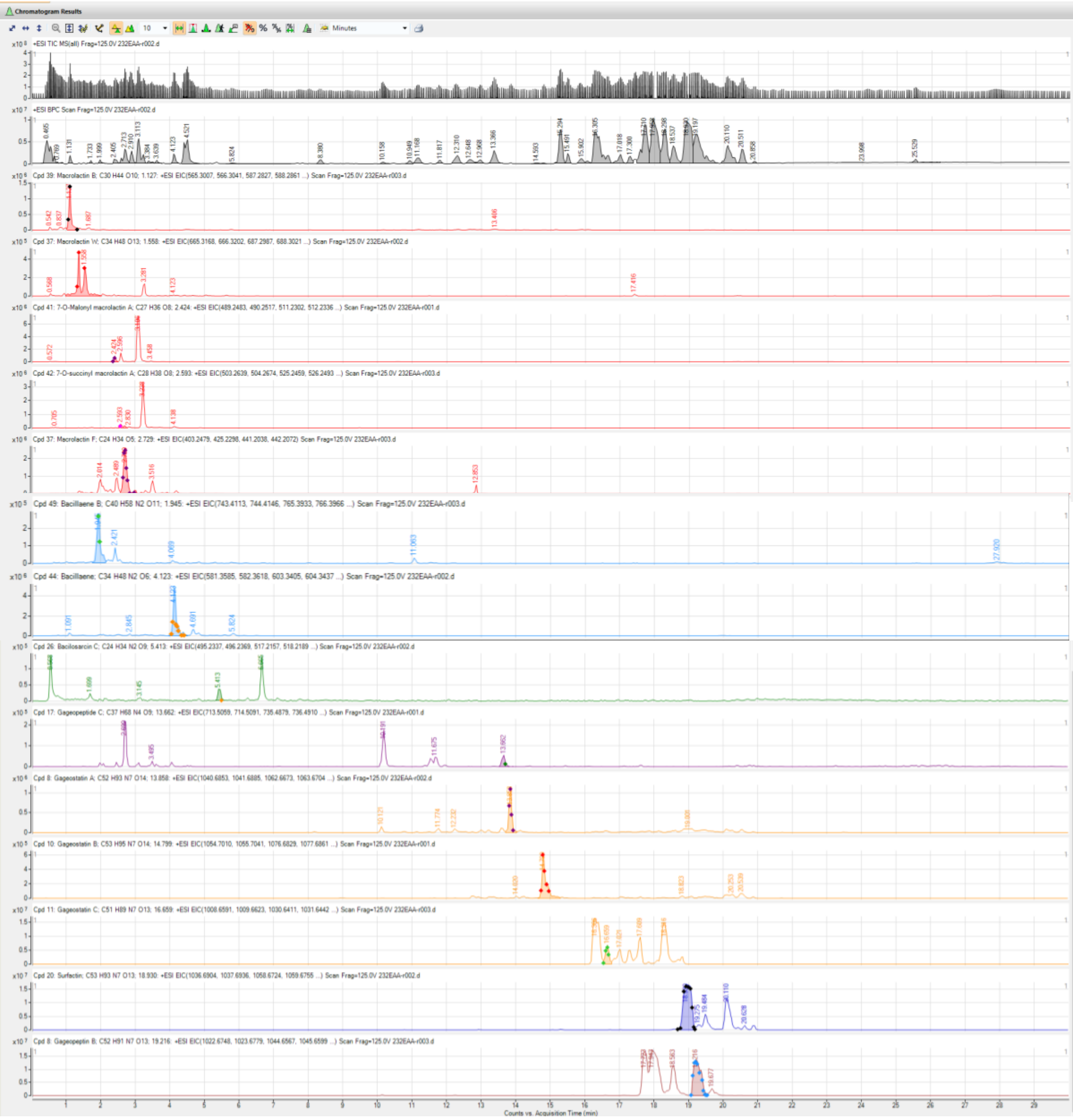

Supplemental Figure S1. Isolate #232 Culture Filtrate Contained Compounds Purified by Ethylacetate Liquid Ex-traction analyzed by LC-QTOF-MS/MS. Ethyl Acetate Liquid Extractions of #232 Culture Filtrate Analyzed LC-QTOF-MS/MS with total ion chromatogram (TIC) and base-peak chromatogram (BPC) in black. LC-QTOF-MS/MS identified compounds from ethyl acetate extracts of #232 culture filtrate with extracted ion chromatograms (EIC) that matched Bacillus subtilis specific compounds including macrolactin compounds (red) bacilosarcin C (green), gageopeptide C (purple), gageostatin compound (orange), surfactin (dark blue), and gageo-peptin B (brown).
