# Supplementary material for: Identification of Pseudomonas protegens and Bacillus subtilis Antimicrobials for Mitigation of Fuel Biocontamination
Source: Biomolecules. 2025 Feb 4;15(2):227. doi: 10.3390/biom15020227 (PMC11853459; doi:10.3390/biom15020227)
Supplement: Supplementary file 1 [file biomolecules-15-00227-s001.zip › 20250122SupplementalFigure S2.pdf]

Surfactin	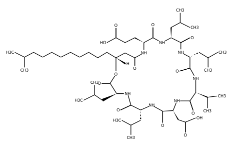	Bacillaene B	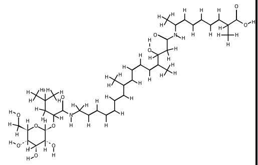
Macrolactin W	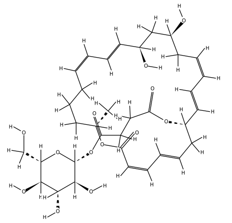	Bacillaene	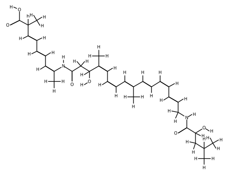
Macrolactin F	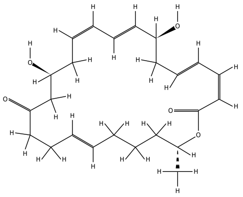	Gageostatin C	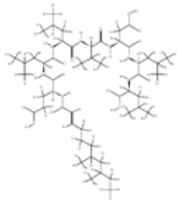
Macrolactin B	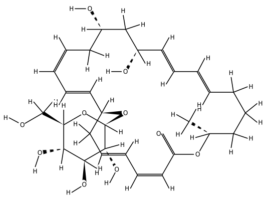	Gageostatin B	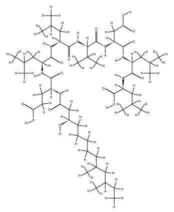
7-O-succinyl macrolactin A	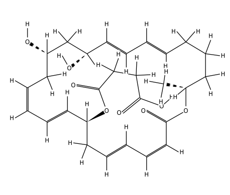	Gageostatin A	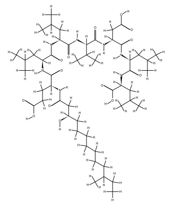
7-O-Malonyl macrolactin A	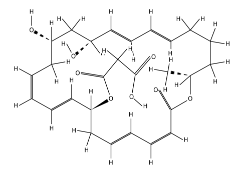	Gageopeptin B	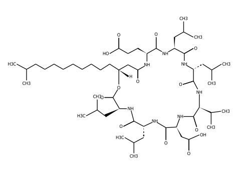
Gageopeptide C	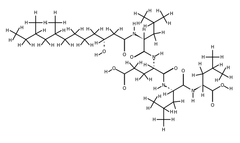	Bacilosarcin C	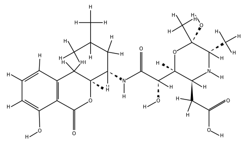

Supplemental Figure S2. Bacillus subtilis Specific Compounds Identified by LC-QTOF-MS/MS in Fuel Isolate #232.
