# Supplementary material for: Identification of Pseudomonas protegens and Bacillus subtilis Antimicrobials for Mitigation of Fuel Biocontamination
Source: Biomolecules. 2025 Feb 4;15(2):227. doi: 10.3390/biom15020227 (PMC11853459; doi:10.3390/biom15020227)
Supplement: Supplementary file 1 [file biomolecules-15-00227-s001.zip › 20250123biomolecules-3353056 Supplementaldatadescription.pdf]

biomolecules

Supplemental Data

In the attached excel files:

Supplemental Data S0: List of All 496 Microbial Fuel Isolates Tested with Agar Plug Screenings against *Pseudomonas putida.*All 496 microbial fuel isolates that were tested as agar donor plugs. Radius of inhibition zones (mm) is given in numbers and displayed by a green heat map with darker green representing larger inhibition zones in all spreadsheets.

Sheet: All 496 Isolates

The spreadsheet contains all 496 microbial fuel isolates tested as agar donor plugs with ID, strain identifier (Name) as tested by species characteristic 16S (bacteria) or 18S (fungi) ribosomal RNA qPCR, and microbial classification (Classification). Results from all 496 microbial fuel isolates agar donor plugs tested against *P. putida.*

Sheet: Top 89 Isolates

Data of the top 89 microbes with proven inhibition zones tested as donor plugs with ID, strain identifier (Name) and microbial classification (Classification). Results from the top 89 microbial fuel isolates agar donor plugs tested against *P. putida.*, *Gordonia* sp., *Y. lipolytica, and H. resinae* is displayed.

Sheet: Top 18 Isolates

Data of the top 18 isolates with proven inhibition zones were tested as donor plugs with ID, strain identifier (Name) and microbial classification (Classification). Results from agar donor plugs testing against the following microbes is displayed: *P. putida, A. venetianus, H. effusa, E. coli* Jm109, *Gordonia* sp., *R. equi, N. luteus, B. atrophaeus, Y. lipolytica, M. guilliermondii, C. ethanolica, H. resinae, A. versicolor, and F. oxysporum.*

Sheet: Top 9 Isolates

Data of the top 9 isolates with proven inhibition zones were tested as donor plugs with ID, strain identifier (Name) and microbial classification (Classification). Results from agar donor plugs testing against the following microbes is displayed: *P. putida, A. venetianus, H. effusa, E. coli* Jm109, *Gordonia* sp., *R. equi, N. luteus, B. atrophaeus, Y. lipolytica, M. guilliermondii, C. ethanolica, H. resinae, A. versicolor, and F. oxysporum.*

Supplemental Data S1: Isolate #232 Culture Filtrate Contained Compounds Purified by Ethyl Acetate Extraction Analyzed by LC-QTOF-MS/MS. Table contains data spreadsheet derived from LC-QTOF-MS/MS analysis under auto MS/MS setting. Data are derived from MassHunter Data Acquisition Software (version 10.0, Agilent, Santa Clare, CA, USA). Data analysis was performed using program tools Agilent Profinder for raw file spectrum alignment followed

by Mass Profiler Professional statistical analysis including identification of unique compounds by subtracting compounds present in the M9 minimum medium control followed by compound identification with established PCDL libraries from *Bacillus subtilis*, and the METLIN 3.1.5 lipid library (G6825AA METLIN, Agilent, Santa Clare, CA, USA). Data display library aligned predicted compounds (Compound); compound formula (Formula); calculated compound mass (Mass); retention time when compound was observed (Retention Time); program-predicted percent match of compound formula with mass spectrometry detected ion mass (Score (Tgt)); program-derived annotations (Annotations); compound algorithm (Compound Algo); frequency of measured primary ion (Frequency); program predicted compound ion species (Ion Species); Ionization mode; MS1 Composite Spectrum; and program-predicted alignment score with METLIN library (Metlin Library Score). Abundance of MS based ion spectrum and replicates for ethyl acetate extracted sample (232 EAA-r0001, -r002, -r003) and M9 medium control (M9 -r0001, -r002, -r003) is shown in heatmap with highest values in red and lowest values in blue.

Supplemental Data S2: Isolate #133 Culture Filtrate Contained Compounds Purified by Ethyl Acetate Extraction Compared to Lipid Extracts from *Pseudomonas aeruginosa* PAO1 and Commercial Pyochelin Analyzed by LC-QTOF-MS/MS. Table contains data spreadsheet derived from LC-QTOF-MS/MS analysis under auto MS/MS setting. Data are derived from MassHunter Data Acquisition Software (version 10.0, Agilent, Santa Clare, CA, USA). Data analysis was performed using program tools Agilent Profinder for raw file spectrum alignment followed by Mass Profiler Professional statistical analysis including identification of unique compounds by subtracting compounds present in the M9 minimum medium control followed by compound identification with established PCDL libraries from *Pseudomonas aeruginosa*, and the METLIN 3.1.5 lipid library (G6825AA METLIN, Agilent, Santa Clare, CA, USA). Data display library aligned predicted compounds (Compound); compound formula (Formula) calculated compound mass (Mass); retention time when compound was observed (Retention Time); program-predicted percent match of compound formula with mass spectrometry detected ion mass (Score (Tgt)); program-derived annotations (Annotations); Compound algorithm (Compound Algo); frequency of measured primary ion (Frequency); program-predicted compound ion species (Ion Species); Ionization mode; MS1 Composite Spectrum; and program-predicted alignment score with METLIN library (Metlin Library Score).

Sheet: #133 Unique Compounds 1019

Lists unique compounds identified in #133 culture filtrate following ethyl acetate lipid extraction in 1019 experiment.

Abundance of MS based ion spectrum and replicates for ethyl acetate extracted sample (133EAA -r0001, -r002, -r003. r004) and M9 medium control (M9 -r0001, -r002, -r003) is shown in heatmap with highest values in red and lowest values in blue.

Sheet: #133 Unique Compounds 0718

Lists unique compounds identified in #133 culture filtrate following ethyl acetate lipid extraction compared to commercial pyochelin in 1019 experiment. Abundance of MS based ion spectrum and replicates for ethyl acetate extracted sample (133 EAA -r0001, -r002, -r003), replicates for commercial pyochelin (pyochelin commercial-r001, -r002, -r003) and M9 medium control (M9 -r0001, -r002, -r003) is shown in heatmap with highest values in red and lowest values in blue.

Sheet: #133 vs PAO1

Lists unique compounds identified in #133 culture filtrate following ethyl acetate lipid extraction compared to lipid extracts from *Pseudomonas aeruginosa* PAO1. Abundance of MS based ion spectrum and replicates for ethyl acetate extracted sample (133EAA- r0001, -r002, -r003), replicates for lipid extracts from *P. aeruginosa* PAO1 (PAO1-r001, r002, r003) and M9 medium control (M9 -r0001, -r002, -r003) is shown in heatmap with highest values in red and lowest values in blue.

represent 100 μM.
