# Supplementary material for: Identification of Pseudomonas protegens and Bacillus subtilis Antimicrobials for Mitigation of Fuel Biocontamination
Source: Biomolecules. 2025 Feb 4;15(2):227. doi: 10.3390/biom15020227 (PMC11853459; doi:10.3390/biom15020227)
Supplement: Supplementary file 1 [file biomolecules-15-00227-s001.zip › 202501Supplemental Figure S3.pdf]

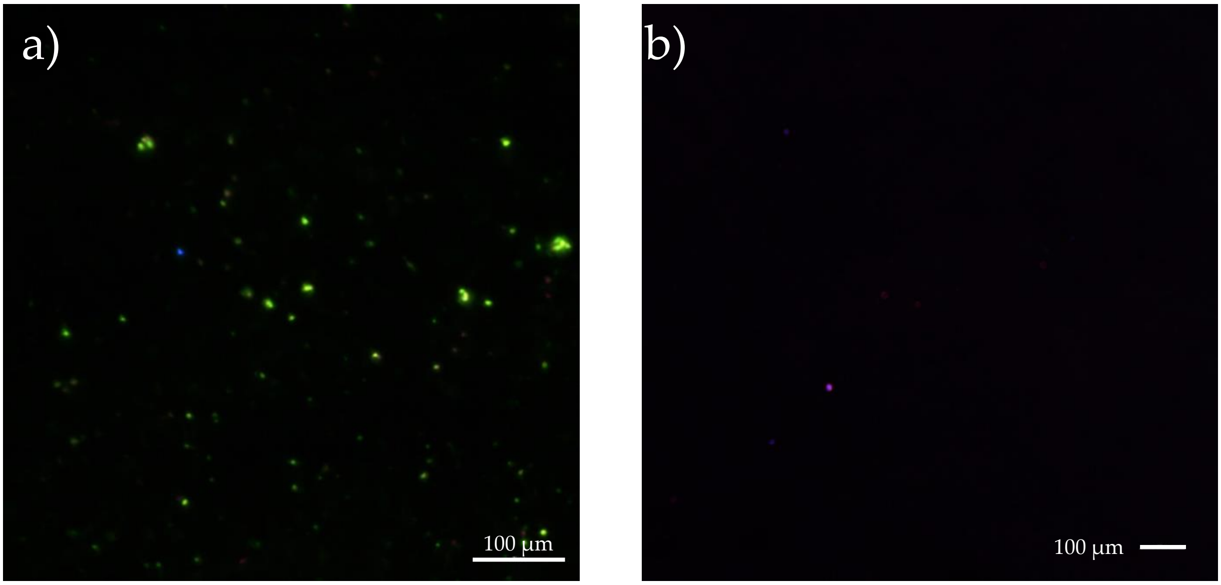


**Supplemental Figure S3.** Antimicrobial Effect of #133 Culture Filtrate on *Gordonia* sp. in Jet Fuel Culture. Confocal microscope images were taking following 2-days incubation of *Gordonia* sp. microbes with 2.5 mL culture filtrate grown for 2 days in Jet A fuel. Samples were derived from the same sample stock that was used in FCM experiment as a cell viability reference control. SYTO 9 staining representative for viable cells shown in green. **a)** Un-exposed *Gordonia* sp. control culture, and **b)** culture exposed to #133 culture filtrate at 2.5 mL (6.9 mg/mL total con-centration) with no viable cells observed. Size bars represent 100 μM.
